# Experience of sibling death in childhood and risk of psychiatric care in adulthood: a national cohort study from Sweden

**DOI:** 10.1007/s00787-019-01324-6

**Published:** 2019-04-01

**Authors:** Mikael Rostila, Lisa Berg, Jan Saarela, Ichiro Kawachi, Anders Hjern

**Affiliations:** 1 Department of Public Health Sciences, Stockholm University, Stockholm, Sweden; 2 Centre for Health Equity Studies, Stockholm University/Karolinska Institutet, 10691 Stockholm, Sweden; 3 Demography Unit, Åbo Akademi University, Turku, Finland; 4 Harvard T.H. Chan School of Public Health, Boston, USA; 5 Clinical Epidemiology, Department of Medicine, Karolinska Institutet, Stockholm, Sweden

**Keywords:** Bereavement, Sibling, Sweden, Grief, Death

## Abstract

Studies have found that sibling loss is associated with an increased risk of death from external causes (i.e. suicides, accidents and homicides). Increased psychiatric health problems following bereavement could underlie such an association. We studied the influence of sibling loss during childhood on psychiatric care in young adulthood, adjusting for psychosocial covariates shared by siblings in childhood. A national cohort born in Sweden in 1973–1982 (*N* = 701,270) was followed prospectively until 2013. Cox proportional hazards models were used to analyse the association between sibling loss during childhood and psychiatric inpatient and outpatient care identified by the Hospital Discharge Register. After adjustment for confounders, the HRs of psychiatric care in men who experienced sibling loss were 1.17 (95% CI 1.07–1.27) while the associations turned non-significant in women after adjustment for family-related psychosocial covariates, HR 1.07 (95% CI 0.99–1.16). An increased risk was found in men bereaved in early childhood (1.22 95% CI 1.07–1.38) and adolescence (1.27 95% CI 1.08–1.48). Among women, loss of a sibling during adolescence was significantly associated with psychiatric care (1.19 95% CI 1.03–1.36). Increased psychiatric health problems following bereavement could underlie the previously found association between sibling loss and mortality from external causes. Family-related psychosocial conditions shared by siblings in childhood may account for the association between sibling death and psychiatric care in adulthood.

**Electronic supplementary material:**

The online version of this article (10.1007/s00787-019-01324-6) contains supplementary material, which is available to authorized users.

## Introduction

Stressful life events, such as losing a family member, are associated with increased risk of several types of health problems, diseases and causes of death among bereaved family members [1–5]. More than 1% of all Swedish children will experience the loss of a sibling prior to their 18th birthday [6]. Health consequences as a result of sibling loss have received less attention than other types of bereavement in the previous literature [7–9]. The social relationship between siblings represents one of the most important and intimate relationships people maintain during childhood and adolescence. Losing a sibling in childhood may be considered an especially unexpected and traumatic event, and the level of grief and its consequences could, therefore, be comparable to that of losing a parent [10–12].

Several studies have found that losing a sibling in adulthood is associated with an increased risk of all-cause and cause-specific mortality [7, 13–15]. Two studies based in Nordic countries have found that the loss of a sibling in childhood exerts an influence on adult mortality risk and that risks are more pronounced for deaths from external causes such as suicides, homicides and accidents [6, 16] which indicates underlying mental and psychiatric health problems. However, it still remains unclear whether sibling loss is associated with an increased risk of such health problems. Lingering grief may contribute to poor mental health, depression, and post-traumatic stress and increase the risk for suicide and accidents. A recent study found that bereaved siblings had higher rates of mental disorders than control siblings after adjustment for pre-existing mental disorders [17]. Other smaller-scale studies found that the loss of a sibling in childhood is associated with psychosomatic health problems such as behavioural problems, emotional disturbances, depression, sleeping difficulties and somatic symptoms such as abdominal pain, stomach aches, headaches, asthma, convulsive states and ulcerative colitis [10, 18, 19]. Yet, few studies have been able to study long-term psychiatric consequences stemming from sibling loss using large-scale total population data including diagnoses for in- and outpatient care.

This study aims to study whether sibling loss is associated with psychiatric health problems in bereaved siblings. We will do so by studying the association between sibling loss in childhood (0–18 years) and risk of in- and outpatient care with an underlying psychiatric diagnosis in adulthood. The fact that siblings share many environmental and familial exposures during childhood increases the risk of confounding. Our analyses will, therefore, consider several family-related psychosocial covariates shared by siblings in childhood. Previous research has suggested that the consequences of bereavement could be more detrimental for certain age groups [6] as well as whether the death was sudden and unexpected [20, 21]. Furthermore, since few empirical large-scale studies have examined consequences of sibling bereavement in men and women separately, knowledge on whether health effects by sibling death in childhood vary by sex is limited. Hence, we will focus on potential differences in associations across different developmental phases of childhood, causes of death and between sexes.

## Methods

This study was performed using information from Swedish national registers. These registers contain data with high validity and low attrition rates [22, 23]. Linkage of different registers is made possible through the unique personal identity number assigned to all Swedish residents at birth or at time of immigration. Random reference numbers replace the personal identity numbers before data are made available for researchers and data are analysed anonymously. Ethical approval was obtained by the ethics committee in Stockholm (no. 2014/415-31/5).

The Medical Birth Register was used to identify all individuals born during 1973–1982 and the study population included all individuals who had at least one full sibling, and who were alive and residing in Sweden during the year of their 18th birthday (*n* = 701,270). The Multi-Generation Register was used to identify parents and full siblings of the index persons. The study population was followed prospectively in the Hospital Discharge Register until they were aged 31–40 years. Individuals with experience of parental death before age 18 were not included.

### Sibling death during childhood

The Cause of Death Register was used to retrieve information on sibling’s time and cause of death. Sibling death was defined as the death of a “live-born” sibling (i.e. excluding still born) before the index person’s 18th birthday. Deaths were categorized as deaths caused by natural causes, i.e. deaths caused by diseases (ICD-8 code: 0000-7969; ICD-9 code: 000-796; and ICD-10 code: A00-R99), and external causes, i.e. deaths caused by accidents, homicide or suicides (ICD-8 code: 8000-9999; ICD-9 code: 800-999; and ICD-10 code: V01-Y98) according to the International Classification of Diseases (ICD).

### Psychiatric care

Psychiatric care was defined as at least one visit or hospital admission (i.e. out- and inpatient care) with a main or complimentary diagnosis with an ICD10 code F00-99 registered in the Hospital Discharge Register during the period between 2002 and 2013. The year of 2002 was chosen as starting point since this is the 1st year where information on outpatient care was available.

### Sociodemographic and parental psychosocial covariates

Information on sex, year of birth, geographic residency when the index person was 18 years old, and parental country of birth was retrieved from the Total Population Register. Information on parent’s highest educational level was based on the parent with the highest level of education (information from the year of the child’s 17th birthday). An indicator of whether or not the household had received social welfare (i.e. if the household had obtained economic assistance of any amount) was retrieved for the year of the child’s 17th birthday. Information on parental education and social welfare benefits was retrieved from the longitudinal integration database for health insurance and labour market studies. Information on whether the parents still lived together when the index child was 17 years old was also retrieved from this database.

Psychiatric disorders in parents were defined as hospitalizations with a diagnosis indicating psychiatric disorders and/or self-inflicted injuries, according to the Hospital Discharge Register. Substance abuse was defined using hospital admissions with an ICD diagnosis indicating alcohol or illicit drug use. Severe criminality was defined as having been convicted of a crime that resulted in a sentence of probation, prison or psychiatric care, according to the Register of Court Convictions. Parental psychiatric and substance abuse disorders and severe criminality were defined as at least one recorded case when the child was aged 0–18 years and was constructed separately for mothers and fathers.

### Statistical analyses

Hazard ratios (HRs) and corresponding 95% confidence intervals (CI) for in- and outpatient psychiatric care for individuals with experience of sibling death during childhood in comparison with individuals without experience of sibling death, were estimated using Cox proportional hazards models. Age was the underlying scale. Person time of follow-up was accumulated from January 2002 until the date of a first admission, date of death according to the National Cause of Death Register or end of follow-up in December 2013. In the multivariate analyses, data were analysed in three different models where the first model was adjusted for year of birth, and the second model additionally for geographic residency, parental country of birth, parents’ highest educational level, number of siblings at age 18, family social welfare receipt, and birth order of siblings. The third model was adjusted for shared psychosocial covariates including parental psychiatric disorders, substance abuse and severe criminality. In addition to analyses of the overall association between sibling death during childhood and psychiatric care in adulthood, the age of the index person when the sibling died (0–5 years, 6–11 years and 12–18 years) were investigated. Additional analyses were also performed comparing natural causes of death with external causes of death.

All analyses were conducted using SAS version 9.4 (SAS Institute, Inc, Cary, North Carolina, USA).

## Results

In the study population, 7986 (1.14%) men and women had experienced the death of a sibling during childhood. The majority of the sibling deaths were from natural causes (73%) and infant deaths (0–1 years) comprised almost half (49%) of all sibling deaths. Differences between the bereaved and non-bereaved groups with regard to sociodemographic characteristics were small, except that bereaved siblings were more likely to have more than two siblings (Table 1). Higher levels of psychiatric and substance abuse disorders and criminal offending were seen for parents of the bereaved compared with the non-bereaved individuals. During follow-up, 5.2% of the bereaved men and 5.9% of the bereaved women received inpatient hospital care for a psychiatric disorder, compared with 4.0% and 4.8% among non-bereaved men and women. Outpatient care for psychiatric disorder was also more common among bereaved men (12.0%) and women (15.2%), compared with men (9.8%) and women (13.7%) in the reference group.

**Table 1:** Characteristics of the study population, born in Sweden during 1973–1982

	Non-bereaved	Bereaved			
		Index person age at time of sibling death			
		Ages 0–18	0–5 years	6–11 years	12–18 years
Characteristic (%)					
No. of individuals (%)	693,284 (98.9)	7986 (1.14)	3527 (0.50)	2164 (0.31)	2295 (0.33)
Sex					
Men	51.8	52.4	52.2	53.4	51.8
Women	48.2	47.6	47.8	46.6	48.2
Sibling age at death (years)					
0–1	–	48.6	76.2	47.9	7.1
> 1	–	51.4	23.8	52.1	92.9
Sibling cause of death					
Natural	–	73.4	88.2	74.4	49.9
External	–	26.5	11.8	25.6	50.2
Number of siblings					
1	55.2	16.2	11.0	13.6	26.6
2	32.0	37.1	39.8	31.9	37.7
> 2	12.8	46.8	49.2	54.5	35.8
Parental country of birth					
Sweden	87.1	83.6	85.0	84.7	80.3
Mixed	8.3	4.3	8.5	8.2	11.4
Other European	3.3	2.9	3.9	3.7	5.5
Non-European	1.4	9.2	2.6	3.4	2.8
Parent’s highest education					
Compulsory school	12.4	16.4	15.4	17.0	17.7
Secondary school	48.7	49.6	48.5	49.2	51.6
University	38.9	34.0	36.1	33.8	30.8
Geographic residency					
City	29.4	26.8	27.9	26.9	25.1
Town	49.0	48.2	47.1	48.3	49.8
Rural	21.7	25.0	25.0	24.9	25.2
Indicators of psychosocial problems in the family					
Mother psychiatric disorder	3.3	5.0	4.1	5.6	5.8
Mother substance abuse	0.5	0.7	0.6	0.8	0.9
Mother severe criminality	0.2	0.2	0.1	0.2	0.4
Father psychiatric disorder	2.7	3.9	3.5	3.9	4.4
Father substance abuse	1.6	2.6	2.5	2.3	2.9
Father severe criminality	2.9	4.7	4.7	3.7	5.7

Hazard ratios (HR) for psychiatric care in relation to sibling death are presented in Tables 2 (men) and 3 (women). HR of psychiatric care for bereaved versus non-bereaved men was 1.25 (95% CI 1.15–1.536). HRs were attenuated after adjustment for sociodemographic factors and psychosocial covariates (HR 1.17, 95% CI 1.07–1.27). An increased risk of psychiatric care was mainly seen for sibling loss before school age (0–5 years); HR 1.22 (95% CI 1.07–1.38) and adolescence (12–18 years); HR 1.23 (95% CI 1.06–1.43), in fully adjusted models. HR of psychiatric care was 1.13 (95% CI 1.04–1.22) in bereaved women, after adjustment for birth year. HRs were attenuated after adjustment for sociodemographic factors (Model 2) and turned statistically non-significant (HR 1.07, 95% CI 0.99–1.16) after adjustment for psychosocial covariates (Model 3). For women, an increased risk of psychiatric care was mainly seen for sibling loss during adolescence; HR 1.19 (95% CI 1.03–1.36), in fully adjusted models.

**Table 2:** Sibling death in childhood and HRs of psychiatric in- and outpatient care in adulthood (2002–2013) among men

	Men						
	No. of cases	Model^a^		Model 2^b^		Model 3^c^	
		HR	95% CI	HR	95% CI	HR	95% CI
Sibling death							
No sibling death	38,191	1		1		1	
Sibling death during childhood	545	1.25	1.15–1.36	1.20	1.10–1.31	1.17	1.07–1.27
Index person age at time of sibling death							
No sibling death	38,191	1		1		1	
Index person aged 0–5 years	241	1.26	1.11–1.43	1.24	1.09–1.41	1.22	1.07–1.38
Index person aged 6–11 years	136	1.11	0.94–1.32	1.08	0.91–1.27	1.04	0.88–1.23
Index person aged 12–18 years	168	1.38	1.19–1.61	1.27	1.09–1.47	1.23	1.06–1.43

^a^Model 1 is adjusted for year of birth

^b^Model 2 is adjusted additionally for number of siblings, birth order, geographic residency, parental country of birth, parent’s highest educational level and receipt of social welfare

^c^Model 3 is adjusted additionally for parental psychiatric disorder, substance abuse, and severe criminality

**Table 3:** Sibling death in childhood and HRs of psychiatric in- and outpatient care in adulthood (2002–2013) among women

	Women						
	No. of cases	Model^a^		Model 2^b^		Model 3^c^	
		HR	95% CI	HR	95% CI	HR	95% CI
Sibling death							
No sibling death	49,139	1		1		1	
Sibling death during childhood	625	1.13	1.04–1.22	1.10	1.02–1.19	1.07	0.99–1.16
Index person age at time of sibling death							
No sibling death	49,139	1		1		1	
Index person aged 0–5 years	266	1.08	0.95–1.21	1.06	0.94–1.20	1.03	0.91–1.16
Index person aged 6–11 years	156	1.05	0.90–1.23	1.03	0.88–1.21	1.00	0.85–1.17
Index person aged 12–18 years	203	1.29	1.13–1.48	1.23	1.07–1.41	1.19	1.03–1.36

^a^Model 1 is adjusted for year of birth

^b^Model 2 is adjusted additionally for number of siblings, birth order, geographic residency, parental country of birth, parent’s highest educational level and receipt of social welfare

^c^Model 3 is adjusted additionally for parental psychiatric disorder, substance abuse, and severe criminality

To investigate potential differences with regard to different ages during follow-up, the study population was divided into age groups (20–24 years, 25–29 years, 30–34 years and 35–40 years) and age-specific analyses were performed. No notable differences were seen in these analyses: age-specific HRs from fully adjusted models in relation to psychiatric care were 1.14, 95% CI 1.05–1.24 (20–24 years), 1.11, 95% CI 1.05–1.18 (25–29 years), 1.11, 95% CI 1.05–1.18 (30–34 years) and 1.10, 95% CI 1.02–1.18 (35–40 years).

In additional analyses, we investigated the importance of birth order and number of siblings (not shown in table). Among the bereaved children, 57% were older and 43% younger than the sibling who died. HR from analyses where the index child was younger than the sibling who died was 1.16, 95% CI 1.06–1.27 whereas HR in analyses where the index child was older than the sibling who died was less pronounced and not statistically significant. Results from the analyses stratified by number of siblings demonstrate that the associations with psychiatric care in adulthood were seen mainly for bereaved children with one or two siblings, whereas no association was seen for bereaved children with three or more siblings.

We also performed a number of additional sensitivity analyses since this, to our knowledge, is the first study to examine sibling loss during childhood and risk of psychiatric care. First, to investigate whether HRs changed depending on the sibling’s age at death, we analysed infant and non-infant sibling death separately. Infant deaths were further categorized into two groups consisting of deaths occurring during the first 28 days, i.e. neonatal mortality (65% of all infant deaths), and deaths after the first 28 days. The results from the analyses of infant sibling death in relation to psychiatric care indicated no differences between these two groups. Adjusted HRs were 1.14 (95% CI 1.03–1.26) for neonatal mortality and 1.13 (95% CI 0.99–1.30) for deaths after the first 28 days. For non-infant sibling deaths, we also investigated the importance of cause of death on in- and outpatient care. Elevated HRs for inpatient care following the loss of a non-infant sibling from external causes was found among both men (HR 1.22, 95% CI 0.96–1.56) and women (HR 1.23, 95% CI 0.97–1.57). HRs for inpatient care following the loss of a non-infant sibling from natural causes were HR 0.83 (95% CI 0.61–1.13) in men and HR 1.12 (95% CI 0.86–1.45) in women. HR for outpatient care did not vary notably by cause of sibling’s death (results not shown). Furthermore, the analyses of the importance of cause of death suggested that the increased risk for inpatient care following sibling death from external causes was more pronounced among persons bereaved in adolescence (HR: 1.44, 95% CI 1.16–1.78). Second, a dichotomous variable, indicating whether or not the child lived with both parents at the age of 17, was added to the analyses as a proxy of parental separation/divorce and as a potentially mediating variable. Estimates were further attenuated when this variable was added to the fully adjusted models. After inclusion of this variable HR for psychiatric care were 1.14 (1.04–1.24) for men and 1.05 (0.97–1.13) for women. Finally, even though the question of children born into bereaved families is beyond the scope of this study we also performed additional analyses including children who lost a sibling before birth. One could assume there would be no causal effect between losing a sibling before being born and later psychiatric care, but the confounding structure would be similar. We found no significant association between exposure to this type of event and risk of psychiatric care in remaining siblings (results not shown).

## Discussion

This large-scale follow-up study based on Swedish population registers found that the death of a sibling in childhood was associated with an increased risk of psychiatric in- and outpatient care in adulthood in men. We found a slightly increased risk of psychiatric in- and outpatient care in women but the associations were attenuated and statistically non-significant after controlling for shared psychosocial factors within the family. However, the death of a sibling in adolescence (12–18 years) was associated with the risk of psychiatric out-patient care in adulthood among both men and women while an increased risk of in-patient care was found only in women. The risk of in-patient care in adolescents was particularly high among those who lost a sibling from an external cause.

The death of a sibling and the attendant grief process may have a significant impact on the individual’s mental and psychological wellbeing when it involves the loss of a companion and source of emotional support. Parents who lose a child might become preoccupied and absorbed with their own grief process. Under such circumstances, they may be unprepared to respond to the needs of the remaining children and signs of psychiatric health problems [6, 12]. The fact that the social support system primarily focuses on the bereaved parents who lost a child may leave remaining siblings unsupported in their grief process [8, 12]. Such adverse social circumstances might have consequences for the mental health of bereaved siblings in adulthood. However, the fact that we found no association between sibling loss and risk of psychiatric care in bereaved siblings with three or more siblings suggests that larger sibling groups could buffer potential negative consequences by bereavement. It has also been suggested that losing a child produces strain in family relationships, occasionally serious enough to result in parental separation [24, 25]. Since previous research has found that divorce is detrimental for the mental health of children in the family [26], parental divorce may be an “indirect” adverse consequence of exposure to sibling loss. Indeed, our findings suggested that some of the association between sibling death and inpatient care was partly explained by a proxy for parental separation/divorce.

Our findings also indicated some sex differences. The fully adjusted models suggested significant overall associations between sibling loss and psychiatric care in men while associations turned non-significant in women after adjustment for family-related psychosocial covariates. Generally it has been suggested that women appear better equipped than men to cope with unforeseen stress-related events, and that they are less vulnerable to the loss of, for example, a partner through widowhood or divorce [27]. However, it has also been found that women are more vulnerable to sibling loss across the life course [6]. The fact that associations turned non-significant in women after adjustment for other childhood adversities in women could indicate that women might be more influenced by other adverse circumstances than bereavement per se when compared to men. Nevertheless, more research on sex differences in psychiatric health outcomes after childhood sibling bereavement is needed to confirm sex differences and uncover potential explanations.

Our findings could be interpreted within a life course perspective [7–10]. The ‘latency’ model in life course theory suggests an independent effect by early-life exposures on adult health, although exposures under certain sensitive periods in childhood may be particularly important for later-life health [28, 29]. Accordingly, bereavement following sibling death during certain developmental stages (i.e. in adolescence) may leave a permanent scar on the child’s psychological and emotional development [28, 29]. For instance, the death of a child in adolescence may have a more profound impact on bereaved siblings when considering that the attachment between siblings is stronger. Accordingly, we found an association between sibling loss in adolescence and psychiatric care in adulthood among both men and women in this study. The ‘accumulation model’ maintains that disadvantages tend not only to cluster but also to mount up over the life course. In line with this model, exposure to bereavement is part of a broader clustering of adverse exposures in childhood, which accumulate as the child approaches young adulthood and gradually worsens across life [28, 29]. For instance, family-related factors may both precede and determine the death of a sibling, but can also accumulate after the death. Thus, this model suggests that losing a sibling could be considered as one of many different childhood adversities that mutually influence mortality and disease. Finally, the ‘pathway’ model suggests that exposures are linked, through a chain of causation [28, 29]. Accordingly, the death of a sibling and the following grief process could be seen as an initial trigger that sets off a chain of negative events such as parental separation, failing school performance, etc., that ultimately leads to poor health. Our findings cannot be explained by one single life course model; rather they lend support to all of these models. For instance, we found some sex differences in the associations that are difficult to interpret without more detailed life course studies. Sibling loss was associated with both in- and outpatient care in men when exposed before school age or in adolescence while an association was only found in women when exposed in adolescence. Thus, it is important that future studies on sibling bereavement examine whether one or several of these models could explain health consequences by sibling loss across the life course and also potential sex differences. Understanding of whether latency, accumulation, pathway and/or combination of these, accounts for health problems in adulthood could also guide policies to prevent adverse consequences of bereavement. Identifying risks for disease at the beginning of the etiologic process will have greater and longer-lasting public health influence than attempting to reverse pathology [30].

An important threat to causal inference in this area of research is the possibility that the death or psychiatric health of siblings in the same family share a common prior cause, i.e. there is confounding of the relationship by an unobserved variable such shared adverse psychosocial conditions within the family during childhood [6]. Hence, there is a risk that it is not merely bereavement per se that contributes to psychiatric health problems among bereaved persons. A unique possibility of our study was the opportunity to consider shared psychosocial covariates within the family including parental psychiatric disorder, substance abuse, severe criminality and family social welfare receipt in our empirical models. The fact that the associations between sibling death and psychiatric care were attenuated and statistically non-significant after controlling for such psychosocial factors in women could indicate that shared psychosocial conditions could be considered shared common prior causes and confounders.

## Limitations

The use of national registers in this study made it possible to study an entire national cohort with minimal attrition. The use of outpatient and inpatient data combined made it possible for us to consider more severe forms of psychiatric disease with the more common and less severe forms of psychiatric health problems treated in outpatient care. It is well known, however, that many cases of psychiatric disease are treated in primary care only or not at all. This makes it probable that our results to some unknown extent are affected by referral bias. Another limitation is that more detailed individual information is required to uncover the actual causal mechanisms that link siblings’ death with psychiatric care. Such information could also minimise the possibility of omitted variable bias. Ideally, one would like to have access to genetic data, more information on shared childhood social environment and family characteristics and detailed data on personal and relational characteristics, which are unfortunately not included in the registers. An especially important limitation is the lack of information on psychiatric health problems in childhood for the index persons and the siblings. Although our analyses were adjusted for parental psychiatric disorder when the index child was aged 0–18 years it is still possible that family history of psychiatric disease underlies the association between sibling unnatural deaths and risk of psychiatric care. Another shortcoming is the lack of indicators on the quality of the relationship and frequency of contact between siblings, which might relate to the risk for psychiatric health problems. Finally, our data does not fully cover the periods included in the analyses since it is based on a fixed cohort of individuals born in 1973–1982 followed until 2013. Hence, follow-up time varies between birth cohorts. However, we performed a sensitivity test only including individuals born in 1982 and the results were similar to our main findings.

Our findings suggested that sibling death in childhood, and particularly in adolescence, is associated with increased risk of psychiatric care in adulthood. Thus, increased psychiatric health problems following bereavement could underlie the previously found association between sibling loss and mortality from external causes.

## Electronic supplementary material

Below is the link to the electronic supplementary material.

Supplementary material 1 (DOCX 19 kb)

## Abbreviations

CI: Confidence intervals
ICD: International classifications of disease
HR: Hazard ratio
CVD: Cardiovascular diseases
