# Supplementary material for: Experience of sibling death in childhood and risk of psychiatric care in adulthood: a national cohort study from Sweden
Source: Eur Child Adolesc Psychiatry. 2019 Apr 1;28(12):1581–8. doi: 10.1007/s00787-019-01324-6 (PMC6861357; doi:10.1007/s00787-019-01324-6)
Supplement: Supplementary file 1 — Supplementary material 1 (DOCX 19 kb) [file 787_2019_1324_MOESM1_ESM.docx]

**Supplement Table 1.** Sibling death in childhood and HRs of psychiatric inpatient care in adulthood (2002-2013)

|  | MEN | | | | | | | | WOMEN | | | | | | | |
| --- | --- | --- | --- | --- | --- | --- | --- | --- | --- | --- | --- | --- | --- | --- | --- | --- |
|  |  | Model^1^ | | Model 2^2^ | | Model 3^3^ | | |  | Model 1^1^ | | Model 2^2^ | | Model 3^3^ | | |
|  | No. of cases | HR | 95 % CI | HR | 95 % CI | | HR | 95 % CI | No. of cases | HR | 95 % CI | HR | 95 % CI | HR | 95 % CI |  |
| Sibling death |  |  |  |  |  | |  |  |  |  |  |  |  |  |  |  |
|  |  |  |  |  |  | |  |  |  |  |  |  |  |  |  |  |
| No sibling death | 14,271 | 1 |  | 1 |  | | 1 |  | 16,160 | 1 |  | 1 |  | 1 |  |  |
| Sibling death during childhood | 216 | 1.33 | 1.17-1.53 | 1.23 | 1.07-1.41 | | 1.19 | 1.04-1.36 | 225 | 1.24 | 1.08-1.41 | 1.15 | 1.01-1.32 | 1.10 | 0.96-1.25 |  |
|  |  |  |  |  |  | |  |  |  |  |  |  |  |  |  |  |
| *Index person age at time of sibling death* | | | | |  | |  |  |  |  |  |  |  |  |  |  |
| No sibling death | 14,271 | 1 |  | 1 |  | | 1 |  | 16,160 | 1 |  | 1 |  | 1 |  |  |
| Index person aged 0-5 years | 95 | 1.33 | 1.09-1.63 | 1.29 | 1.05-1.58 | | 1.25 | 1.02-1.54 | 84 | 1.02 | 0.82-1.26 | 0.97 | 0.78-1.20 | 0.92 | 0.74-1.14 |  |
| Index person aged 6-11 years | 53 | 1.17 | 0.89-1.53 | 1.08 | 0.83-1.42 | | 1.03 | 0.79-1.35 | 59 | 1.22 | 0.95-1.58 | 1.15 | 0.89-1.49 | 1.10 | 0.85-1.42 |  |
| Index person aged 12-18 years | 68 | 1.50 | 1.18-1.90 | 1.29 | 1.01-1.63 | | 1.24 | 0.98-1.57 | 82 | 1.58 | 1.27-1.96 | 1.43 | 1.15-1.78 | 1.36 | 1.10-1.70 |  |

^1^ Model 1 is adjusted for year of birth.

^2^ Model 2 is adjusted additionally for number of siblings, birth order, geographic residency, parental country of birth, parent’s highest educational level and receipt of social welfare

^3^ Model 3 is adjusted additionally for parental psychiatric disorder, substance abuse, and severe criminality

**Supplement Table 2.** Sibling death in childhood and HRs of psychiatric outpatient care in adulthood (2002-2013)

|  |  | MEN | | | | | |  | WOMEN | | | | | |
| --- | --- | --- | --- | --- | --- | --- | --- | --- | --- | --- | --- | --- | --- | --- |
|  |  | Model^1^ | | Model 2^2^ | | Model 3^3^ | |  | Model 1^1^ | | Model 2^2^ | | Model 3^3^ | |
|  | No. of cases | HR | 95 % CI | HR | 95 % CI | HR | 95 % CI | No. of cases | HR | 95 % CI | HR | 95 % CI | HR | 95 % CI |
| Sibling death |  |  |  |  |  |  |  |  |  |  |  |  |  |  |
|  |  |  |  |  |  |  |  |  |  |  |  |  |  |  |
| No sibling death | 35,030 | 1 |  | 1 |  | 1 |  | 45,863 | 1 |  | 1 |  | 1 |  |
| Sibling death during childhood | 503 | 1.25 | 1.15-1.37 | 1.21 | 1.11-1.32 | 1.18 | 1.08-1.29 | 579 | 1.12 | 1.03-1.22 | 1.09 | 1.01-1.19 | 1.06 | 0.98-1.15 |
|  |  |  |  |  |  |  |  |  |  |  |  |  |  |  |
| *Index person age at time of sibling death* | | | | |  |  |  |  |  |  |  |  |  |  |
| No sibling death | 35,030 | 1 |  | 1 |  | 1 |  | 45,863 | 1 |  | 1 |  | 1 |  |
| Index person aged 0-5 years | 222 | 1.26 | 1.10-1.44 | 1.24 | 1.09-1.42 | 1.22 | 1.07-1.39 | 245 | 1.06 | 0.94-1.21 | 1.05 | 0.93-1.20 | 1.02 | 0.90-1.16 |
| Index person aged 6-11 years | 123 | 1.09 | 0.91-1.30 | 1.06 | 0.89-1.26 | 1.02 | 0.85-1.22 | 143 | 1.03 | 0.87-1.21 | 1.01 | 0.86-1.19 | 0.98 | 0.83-1.16 |
| Index person aged 12-18 years | 158 | 1.41 | 1.21-1.65 | 1.30 | 1.11-1.52 | 1.27 | 1.08-1.48 | 191 | 1.30 | 1.13-1.50 | 1.23 | 1.07-1.42 | 1.19 | 1.03-1.37 |

^1^ Model 1 is adjusted for year of birth.

^2^ Model 2 is adjusted additionally for number of siblings, birth order, geographic residency, parental country of birth, parent’s highest educational level and receipt of social welfare

^3^ Model 3 is adjusted additionally for parental psychiatric disorder, substance abuse, and severe criminality
